# Identification and biodiversity patterns of *Aspergillus* species isolated from some soil invertebrates at high altitude using morphological characteristics and phylogenetic analyses

**DOI:** 10.7717/peerj.15035

**Published:** 2023-03-28

**Authors:** Mohamed Fadl Awad, Bander Albogami, Tarombera Mwabvu, Montaser M. Hassan, Alaa Baazeem, Mohamed M. Hassan, Mohsen Mohamed Elsharkawy

**Affiliations:** 1 Department of Biology, College of Science, Taif University, Taif, Saudi Arabia; 2 High Altitude Research Centre, Taif University, Taif, Saudi Arabia; 3 School of Biology & Environmental Sciences, University of Mpumalanga, Mbombela, South Africa; 4 Department of Agricultural Botany, Faculty of Agriculture, Kafrelsheikh University, Kafr Elsheikh, Egypt

**Keywords:** *Aspergillus* spp., Mycotoxins, *Armadillidium vulgare*, *Porcellio laevis*, Millipedes

## Abstract

**Background:**

The carcinogenic, mutagenic, and teratogenic chemicals such as aflatoxin are a worldwide health problem. *Aspergillus* spp., responsible for most cases of aflatoxin contamination, are common in the environment and spread easily to many different types of food. The objectives of this study were to conduct a survey of fungi associated with three soil invertebrates in Taif, Saudi Arabia, identify these isolates and explore mycotoxins formation.

**Methods:**

In total, 114 fungal isolates were collected from various soil invertebrates (millipedes, *Armadillidium vulgare* and *Porcellio laevis*) in Taif, Saudi Arabia, among them, 22 isolates were identified as *Aspergillus* spp. based on morphological and molecular characteristics followed by both *Fusarium* and *Penicillium.*

**Results:**

The sequences of ITS 1 and ITS 4 were utilized. Using bootstrap analysis, phylogenetic tree was split into two distinct clusters. Five sub clusters were included inside the first major cluster, and their bootstrap value was 99%. While, there were two small clusters in the second major cluster. All the tested *Aspergillus* strains were able to have a single PCR fragment amplified using the primer AspTef. TEF-1 DNA sequence bootstrap analysis with 1,000 replicates revealed two distinct groups. Additionally, the *Aspergillus* isolates were grouped into two different clusters with about 65% genetic similarity using ISSR-PCR analysis. The standard polymerase chain reaction was used to effectively amplify the *Aopks, afl-A* and *omt*-A genes in aflatoxigenic *Aspergillus* strains. Four *Aspergillus* strains used in this investigation were shown to generate aflatoxin B1. While, three *Aspergillus* stains showed ochratoxin genes.

**Conclusions:**

In conclusion, the results indicate significant differences in the fungal community between ecoregions and soil invertebrates. Moreover, mycotoxin detection and identification among *Aspergillus* isolates were elucidated. This study could shed light on the risk of mycotoxin contamination along the supply chain.

## Introduction

The existence of invertebrates is an indicator that area is suitable for the long-term growth of nutritious plants or trees (Stork & Eggleton, 1992). Mycoflora constitutes an important part of the soil ecosystem, playing a fundamental role in the biotic and abiotic interactions in this environment, participating in the recycling of soil nutrients and decomposition of organic matter to make them available to plants (Val-Moraes et al., 2009). Therefore, soil fungi communities contribute to the alleviation of soil degradation and fertility (Jeffries et al., 2003; Treseder et al., 2014; Sterkenburg et al., 2015).

The fungal community of soil is an immensely diverse group of organisms. A recent study on the diversity of soil fungi revealed around 80,500 operational taxonomic units (OTUs) occurring in soils worldwide (Treseder et al., 2014; Rosas-Medina, Macia-Vicente & Piepenbring, 2020). Local environmental variables, such as physical and chemical soil features, influence the diversity of soil fungi (Tardy et al., 2015), which in turn strongly affects the current diversity of fungal communities (Requena et al., 2001). *Aspergillus* is a diverse genus followed by a large number of species, which covers all countries of the world and is found in different climatic conditions, as well as in different soil types (Burrough et al., 2012; Monmi, Nguyen & Lee, 2022). It is also known for its secretion of mycotoxins, as well as its ability to spoil food, in addition, it is known for high pathogenicity to humans and animals (Frisvad et al., 2011; Hussein, El-Said & Yassein, 2020; Wei et al., 2022). Moreover, many species of this fungus are used in the industrial field, biotechnology and used in food fermentation processes such as cheeses as well as the production of many antibiotics, organic acids, medicines, or enzymes (Mazrou et al., 2020).

*Aspergillus* species were classified and defined in the past on the basis of morphological characteristics, but recently the matter has changed when relying on molecular and chemical characterization, which gave a kind of accuracy and credibility in molecular characterization over morphological characterization (Samson et al., 2014; Gautier, Normand & Ranque, 2016). It is now possible to use morphological traits along with molecular identification, which is the case in most recent studies. The th phenotype-based classification of subspecies and divisions is largely consistent with currently published lineages. Recent phylogenetic studies have shed light on the relationships among *Aspergillus* species, but there is still a puzzling question about the relationship between the new nomenclature with a single name for fungi (Tamura, Kawahara & Sugiyama, 2000; Houbraken & Samson, 2011; Houbraken, Vries & de Samson, 2014; Hassan, Farid & Gaber, 2019; Hassan et al., 2022). It is also important to study and determine the evolutionary relationships of species in *Aspergillus* and closely related genera (McNeill et al., 2012; Vesth et al., 2018). Standardized procedures based on extrolite characterization, morphological traits, and multilocus DNA sequence studies are routinely used for identifying *Aspergillus* species (Geiser et al., 2008; Mazrou et al., 2020. Molecular markers used for the identification, characterization and evolution of *Aspergillus* species over time are increasingly being studied. Furthermore, molecular markers used in *Aspergillus* have included the internal transcription spacer sequence (ITS), calodulin (CaM), and *β*-tubulin (BenA), Tef1 gene and RNA polymerase II second largest subunit (RPB2) sequences. The molecular marker is employed for identifying *Aspergillus* species because of the extensive gene database available, the relative simplicity of locus amplification, and genetic polymorphism (Samson et al., 2014; Raja et al., 2017; Monmi, Nguyen & Lee, 2022). These locus are insufficient to identify all *Aspergillus* species correctly, thus secondary identification parameters such as the SCAR marker are needed (Hassan, Farid & Gaber, 2019). About 25 species of *Aspergillus* have been reported from Saudi Arabia (Kamel, 2014; Taha et al., 2021). *A. koreanus*, a previously unknown species of *Aspergillus*, has just been described (Ameen et al., 2022). Recently, six more species have been documented in Saudi Arabia, *A. asperescens* and *A. flavips*, *A. ustus, A. egyptiacus*, *A. versiolor* and *A. terreus* (El-Samawaty et al., 2012). Hence, the objectives of the present work were to isolate, identify, and molecularly characterize the mycological diversity of specific soil invertebrates (millipedes, *Armadillidium vulgare* and *Porcellio laevis*) in Taif, Saudi Arabia, and to explore the mycotoxin gene in the isolated *Aspergillus* species.

## Materials and Methods

### Fungal isolation

Twenty samples of each soil invertebrates (*Armadillidium vulgare*, Millipedes and *Porcellio laevis*) were collected from three regions in Taif Governorate (Hawia, Shafa, and Wady Ghazal). Spores or mycelium fragments on the cuticle surface of carcasses were resuspended by shaking the bodies in water containing 5% Tween, according to Samson et al. (2014). Standard PDA/chloramphenicol medium was used to plate 10 µl of dilutions of this solution. Single germinations (or mycelium regeneration) were moved to a new plate after 6 days in an incubator (25 ± 2 °C). Each insect had only one isolate chosen from a pool of thalli with identical appearance. The morphological stability of these isolates was monitored by repeated purification steps after each of their consecutive transfers. Single spore purification was performed on conidial species, including *Aspergillus* spp.

### Morphological identification of fungal isolates

Pure cultures were grown on Czapek Agar (CZA) medium. Sporulation was induced by subjecting cultures to ultraviolet light. Isolates were characterized according to morphological features, cultural characteristics such as pigmentation of the mycelium and direction of hyphal growth, aerial or lateral, microscopic observation of structures involved in asexual and sexual reproduction (spores) and the presence of fruiting bodies. Identification was accomplished based on the morphology of fungi (Raper & Fennell, 1965; Samson & Pitt, 2000; Pitt & Hocking, 2009; Samson et al., 2014).

### DNA extraction

Mycelia from certain pathogenic fungi were put into Czapex Dox broth and cultured for 5 days at 27 °C in order to harvest their DNA. Using the standard fungal genomic DNA extraction protocol, DNA was isolated from each fungal sample (Al-Samarrai & Schmid, 2000).

### PCR amplification of ITS region and TEF gene

ITS and TEF genes were PCR amplified with the primers listed in Table 1. Reactions of ITS and TEF genes were carried out according to Hassan, Farid & Gaber (2019) and Druzhinina et al. (2005), in 50 µl volume containing 2 µl (20 ng) of genomic DNA, 1 µl of primer (20 p.mol), 25 µl of Go Taq^®^ Green Master Mix (Promega, Madison, WI, USA) deionized distilled water (up to a total volume of 50 µl). The C1000TM Thermo Cycler from Bio-Rad (Hercules, CA, USA) was calibrated for DNA amplification at 94 °C for 10 min before adding Taq polymerase, then for 40 cycles. Each cycle included 1 min at 94 °C, 1.5 min at 58 °C, and 2.5 min at 72 °C, followed by a final extension time of 7 min at 72 °C. The results of DNA amplification were examined using electrophoresis on 2% agarose gel run in TBE. The gels were stained with ethidium bromide (5 µg ml^−1^). DNA Ladder RTU (100 bp), (Gene Direx^®^) was used as a standard. The Bio-Rad Gel Doc 2000 was used to photograph DNA under UV light for visualization.

**Table 1:** Primer sequences and amplicon sizes used in this study.

Primer name	Primer sequence (5′ → 3′)	Product size (bp)
ITS-1	TCC GTA GGT GAA CCT GCG G	600
ITS-4	TCC TCC GCT TAT TGA TAT GC	
Tef-F	CAT CGA GAA GTT CGA GAA GG	700
Tef-R	AAC TTG CAG GCA ATG TGG	
*Omt-AF*	GACCAATACGCCCACACAG	300
*Omt-AR*	CTTTGGTAGCTGTTTCTCGC	
*Aopks-F*	CAGACCATCGACACTGCATGC	549
*Aopks-R*	CTGGCGTTCCAGTACCATGAG	
*AflA-F*	GTAGGGTTCCTAGCGAGCC	497
*AflA-R*	GGAAAAAGATTGATTTGCGTTC	
*Gdh-F*	TTAACCGCGGCCTGCCTGG	900
*Gdh-R*	GGCTGCTGGCCGAACTGACTT	

### Sequencing analysis of ITS region and TEF gene

To get the sequences, we used polymerase chain reaction (PCR) and sequenced the resulting amplicons directly according to Hassan et al. (2022) using a 3130 Genetic Analyzer (Applied Biosystems, Waltham, MA, USA) at Macrogen Co., Seoul, South Korea. Data from the sequencing experiment was compared to those in the GenBank database (http://www.ncbi.nlm.nih.gov/BLAST/), using the nucleotide BLAST program to identify homology between the PCR fragments and sequences in the GenBank database. Using their assigned accession numbers, the sequences were submitted to GenBank at the NBCI (National Center for Biotechnology Information).

### Molecular detection of aflatoxin and ochratoxin-producing genes

To detect the genes responsible for producing aflatoxin and ochratoxin, two published primers were used. Primers’ sequences were tabulated in Table 1. A 25 µl reaction volume was used for the PCR according to Hussein, El-Said & Yassein (2020). The C1000 was used to conduct the reactions. Denaturation at 94 °C for 5 min using a Thermo Cycler (BioRad, Hercules, CA, USA); 30 cycles of 1 min at 94 °C, 1 min at 58 °C, and 1 min at 72 °C; last step is extension at 72 °C for 10 min. PCR products were examined on an ethidium bromide-stained 1.3% agarose gel.

### Inter simple sequence repeats (ISSR)-PCR analysis

For inter simple sequence repeats analysis, PCR of *Aspergillus* isolates was performed according to Mazrou et al. (2020). Reactions of PCR-ISSR were carried out in 25 µl volume and five ISSR primers (ISSR-4, ISSR-5, ISSR-8, ISSR-18, and ISSR-28) were used to amplify the genomic DNA. Primers (Macrogen Inc., Seoul, Korea) used in this analysis are listed in Table 1. An easy matching coefficient determined using Jaccard’s coefficient was used to provide an estimate for the similarity matrix (Rohlf, 2000).

### Data analysis

Version 2 of ClustalW was used for the sequence alignment (Larkin et al., 2007). The haplotype diversity was examined using the DnaSP program (Librado & Rozas, 2009). Phylogenetic analysis and exploratory data were performed using the R Project for Statistical Computing (R Core Team, 2022). Using our own code, the haplotype sequence matrix was used to obtain the haplotype distance matrix (20 × 41). To elucidate the genetic relatedness between the *Aspergillus* isolates and molecular identification, a phylogenetic tree was constructed based on the sequence analysis of the ITS and TEF-1 *α* regions, using the neighbor-joining method in the MEGA 7.1 software.

## Results

### Isolation of *Aspergillus* species

In total, 114 fungal species belonging to 10 genera were isolated from some soil invertebrates (millipedes, *Armadillidium vulgare* and *Porcellio laevis*) in Taif Governorate, Saudi Arabia (Table 2). Twenty-two of them were identified as *Aspergillus* sp. *Aspergillus* was isolated from all soil invertebrate samples. The isolates swiftly expanded their colonies once grown on PDA, and although their mycelium used to start with white and floccose, it eventually became a dark color with black spores. The twenty-two *Aspergillus* isolates locations and the animal’s name is presented (Table 3). Nine *Aspergillus* isolates were isolated from millipedes, six of them (TU-1, TU-2, TU-3, TU-19, TU-20 and TU-23) were isolated from Shafa, Taif, and three of them (TU-14, TU-15 and TU-16) were isolated from Wady Ghazal, Taif, Saudi Arabia. On the other hand, eight *Aspergillus* isolates were isolated from *A. vulgare*. Five isolates (TU-4. TU-5, TU-6, TU-8 and TU-9) were isolated from Hawia, Taif. While, three *Aspergillus* isolates (TU-33, TU-35 and TU-36) were isolated from Shafa, Taif. Finally, five *Aspergillus* isolates (TU-13, TU-24, TU-25, TU-32 and TU-33) were isolated from *Porcellio laevis* in Hawia, Taif, Saudi Arabia.

**Table 2:** A list of fungi isolated from some soil invertebrates at high altitudes in Taif, Saudi Arabia.

Fungi	Source of collection			No. isolates
	Millipedes	*A. vulgare*	*Porcellio laevis*	
*Penicillium* sp.	7	2	6	15
*Alternaria* sp.	3	2	5	10
*Aspergillus* sp.	10	7	5	22
* Basidioascus* sp.	5	0	0	5
*Candida* sp.	4	8	3	15
*Curvularia* sp.	2	0	1	3
*Fusarium* sp.	9	7	5	21
*Trichoderma* sp.	4	6	2	12
*Geotrichum* sp.	5	3	2	10
*Talaromyces* sp.	1	0	0	1
Total	52	37	31	114

**Table 3:** The sources and locations of *Aspergillus* species isolated from soil invertebrates in Taif, Saudi Arabia.

Isolates	Species	Source	Locations
TU-1	*Aspergillus calidoustus*	millipedes	Shafa, Taif
TU-2	*Aspergillus ochraceus*	millipedes	Shafa, Taif
TU-3	*Aspergillus neoflavipes*	millipedes	Shafa, Taif
TU-4	*Aspergillusvenezuelensis*	*A. vulgare*	Hawia, Taif
TU-5	*Aspergillus caespitosus*	*A. vulgare*	Hawia, Taif
TU-6	*Aspergillus caespitosus*	*A. vulgare*	Hawia, Taif
TU-8	*Aspergillus venezuelensis*	*A. vulgare*	Hawia, Taif
TU-9	*Aspergillus flavipes*	*A. vulgare*	Hawia, Taif
TU-13	*Aspergillus caespitosus*	*Porcellio laevis*	Hawia, Taif
TU-14	*Aspergillus caespitosus*	millipedes	Wady Ghazal, Taif
TU-15	*Aspergillus flavipes*	millipedes	Wady Ghazal, Taif
TU-16	*Aspergillus europaeus*	millipedes	Wady Ghazal, Taif
TU-19	*Aspergillus ustus*	millipedes	Shafa, Taif
TU-20	*Aspergillus aculeatus*	millipedes	Shafa, Taif
TU-23	*Aspergillus oryzae*	millipedes	Shafa, Taif
TU-24	*Aspergillus flavus*	*Porcellio laevis*	Hawia, Taif
TU-25	*Aspergillus neoflavipes*	*Porcellio laevis*	Hawia, Taif
TU-31	*Aspergillus arcoverdensis*	*Porcellio laevis*	Hawia, Taif

### Morphological identification

**Figure 1:**
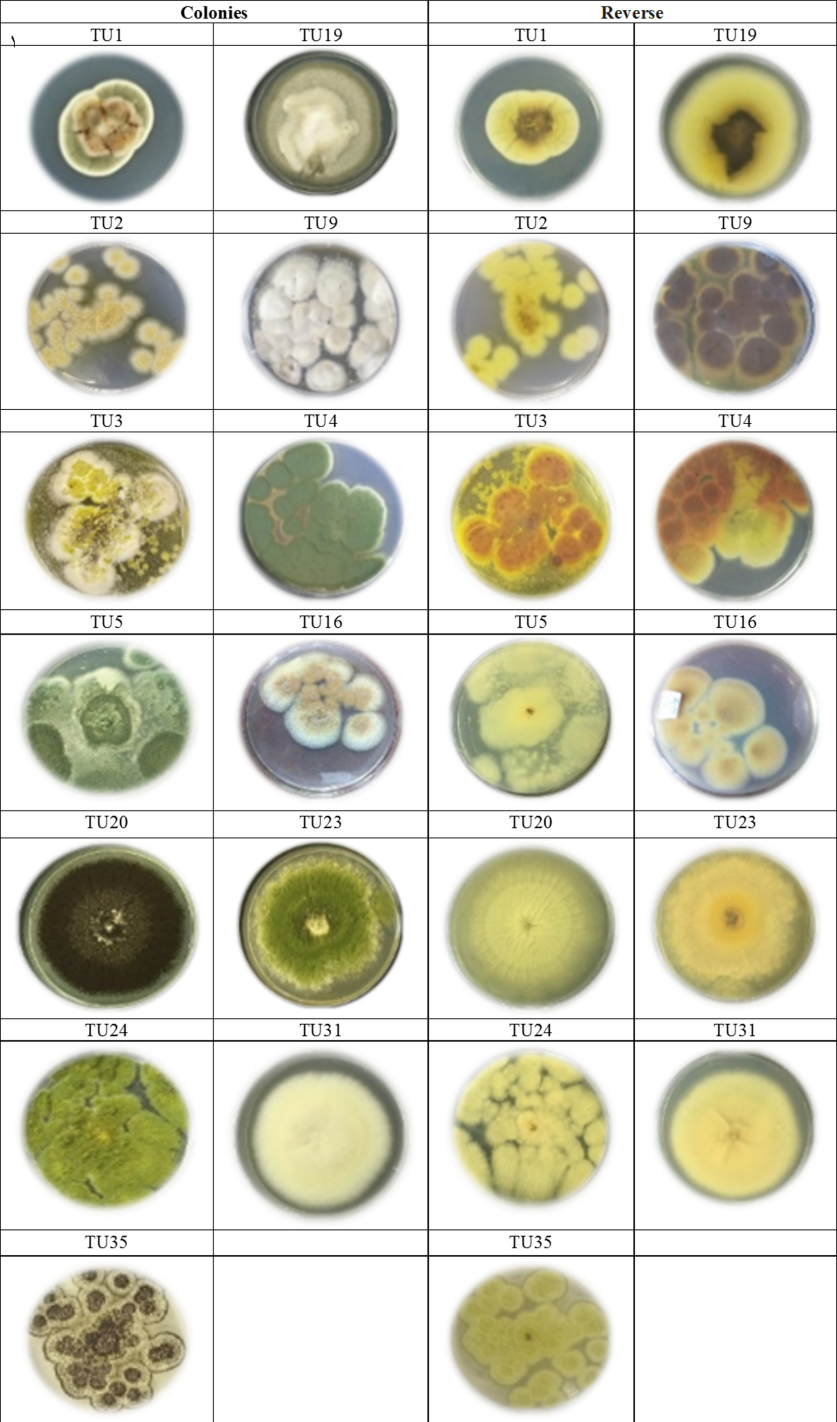
Cultures of *Aspergillus.* species isolated from soil invertebrates in Taif, Saudi Arabia on Czapek Agar (CZA) medium after 6 days at 25 ± 2 °C.

**Figure 2:**
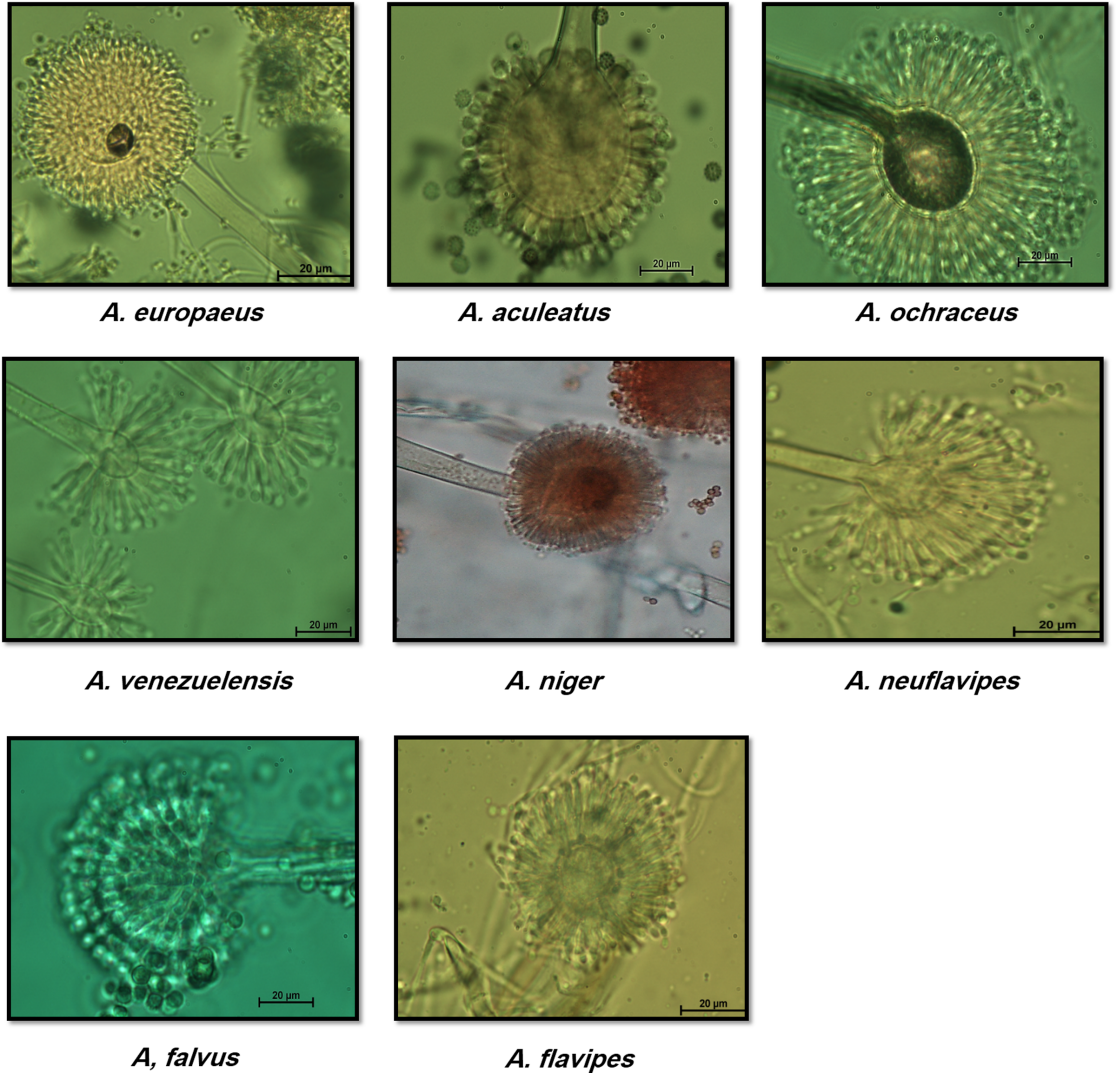
Morphological characteristics of of *Aspergillus* species isolated from soil invertebrates in Taif, Saudi Arabia on Czapek Agar (CZA) medium after 6 days at 25 ± 2 °C.

Two predominated *Aspergillus* sections were isolated, Flavipedes and Nidulantes (Table 3). The morphological characteristics of *Aspergillus* species are shown in Figs. 1 and 2. The colony of *A. ochraceus* TU2 belonging to *Aspergillus* section Circumdati was yellow-gold, granular and had a reddish-brown color on the reverse of the plate. Conidiophore was yellowish pale brown and coarsely rough. Vesicle was biseriate, globose and entirely Metula. While, the isolates *A. europaeus* TU16, 32 and 36 belonging to *Aspergillus* section Cremei were yellowish grey, velutinous to floccose, colorless, and light yellow on the reverse of the plate. Conidiophore was colorless and delicately roughened. Vesicle was biseriate, pyriform or globose and Metula covering 3/4 ∼the entire vesicle, globose, subglobose, elliptical conidia and coarsely roughened (Fig. 2). Sclerotia and Hülle cells were absent. *A. flavus* TU24 and *A. oryzae* TU24 were categorized under *Aspergillus* section Flavi. Their colonies were granular, floccose, yellowish green to olive green, and colorless to yellow, pale yellow color on the reverse of the plate, respectively. Conidiophore was pale brown, colorless and rough. Vesicle was biseriate and biseriate, rarely uniseriate, globose, subglobose and Cleistothecia present or not ascomata, respectively. Two isolates (*A. flavipes* TU9&15 and *A. neoflavipes* TU3&25) belong to *Aspergillus* section Flavipedes were floccose to cottony, velutinous, white to yellowish white, brightly to moderate yellow and pale yellowish orange and strong yellow to medium olive brown on the reverse of the plate, respectively. Conidiophore was hyaline to light brown, very pale brown and smooth. Vesicle was biseriate, rarely uniseriate and biseriate, spathulate, sometimes pyriform and subglobose, pyriform or spathulate and covering one-half to two-thirds of the vesicle. On the other hand, the colonies of *Aspergillus arcoverdensis* TU31&33, belonging to *Aspergillus* section Fumigati, were white to orange, white, floccose, and yellowish white to pale orange color on the reverse of the plate. Conidiophore was hyaline to pale yellowish brown and smooth. Vesicle was uniseriate, subglobose to hemispherical, and covering the upper half of the vesicle Metula. *Aspergillus* section Nidulantes was represented by *A. caespitosus* TU5,6,13&14 and *A. venezuelensis* TU4&8. Colony was velvety, graysih green, and floccose at center, velvety at edges or light yellow at center, white at edge and vinaceous buff to grey olivaceou, light yellow fading into cream white color on the reverse of the plate. Conidiophore was pale brown or brown to yellow and smooth. Vesicle was biseriate, hemisphere to subclavate and subglobose, pyriform, covering the upper half or two-thirds of the vesicle Metula. Conidia were globose or globose to subglobose and echinulate, spinulose. Ascomata, ascospores, globose and Hülle cells were present. On the other side, *Aspergillus aculeatus* TU20 and *A. niger* TU35, belonging to *Aspergillus* section Nigri, were dark brown/gray tones or dark brown to black, velvety, granular, and pale to yellow, colorless to light yellow color on the reverse of the plate. Conidiophore was slightly brown and smooth. Vesicle was uniseriate and biseriate, spherical, globose, and entirely Metula. Conidia were ellipsoid, spiny and globose or ellipsoid and very rough irregular. Fruiting bodies were absent. While, *Aspergillus* section Usti was represented by *A. calidoustus* TU1 and *A. ustus* TU19. Colony was floccose, blond/greyish yellow and yellow with beige or olive-brown center or yellow-olive edge with olive brown center color on the reverse of the plate. Conidiophore was brown and smooth. Vesicle was biseriate, pyriform to broadly spathulate and hemispherical to subglobose, covering the upper half to entire surface of the vesicle Metula. Conidia were globose, and very rough. Hülle cells were sparsely produced.

### Molecular identification of *Aspergillus* species using 5.8S-ITS region sequence

To perform nucleotide sequencing, genomic DNA was successfully isolated from all *Aspergillus* isolates. All of the *Aspergillus* isolates utilized in this analysis had their rDNA internal transcribed spacer (ITS) regions amplified using the universal primers ITS 1 and ITS 4. The acquired sequences were submitted to NCBI for BLAST analysis in order to determine the identification of the isolates (accession numbers ON160831 –ON160852). The length of the ITS sequences varied greatly among *Aspergillus* isolates, from 550 to 650 bp. There was almost perfect nucleotide sequence homology between *Aspergillus* isolates (Table 4).

**Table 4:** The NCBI BLAST query of ITS region sequences for *Aspergillus* species isolated from invertebrates in Taif, Saudi Arabia.

Isolates	Species	Query coverage %	*E* value	Identity%	Accession number
TU-1	*Aspergillus calidoustus*	98.00	0.00	99.00	ON160831
TU-2	*Aspergillus ochraceus*	100.00	0.00	100.00	ON160832
TU-3	*Aspergillusneoflavipes*	100.00	0.00	100.00	ON160833
TU-4	*Aspergillus venezuelensis*	100.00	0.00	100.00	ON160834
TU-5	*Aspergillus caespitosus*	99.00	0.00	99.00	ON160835
TU-6	*Aspergillus caespitosus*	100.00	0.00	100.00	ON160836
TU-8	*Aspergillus venezuelensis*	99.00	0.00	100.00	ON160837
TU-9	*Aspergillus flavipes*	100.00	0.00	100.00	ON160838
TU-13	*Aspergillus caespitosus*	100.00	0.00	99.00	ON160839
TU-14	*Aspergillus caespitosus*	100.00	0.00	100.00	ON160840
TU-15	*Aspergillus flavipes*	100.00	0.00	99.00	ON160841
TU-16	*Aspergillus europaeus*	100.00	0.00	99.00	ON160842
TU-19	*Aspergillus ustus*	100.00	0.00	99.00	ON160843
TU-20	*Aspergillus aculeatus*	99.00	0.00	100.00	ON160844
TU-23	*Aspergillus oryzae*	100.00	0.00	100.00	ON160845
TU-24	*Aspergillus flavus*	100.00	0.00	100.00	ON160846
TU-25	*Aspergillusneoflavipes*	99.00	0.00	100.00	ON160847
TU-31	*Aspergillus arcoverdensis*	100.00	0.00	100.00	ON160848
TU-32	*Aspergillus europaeus*	100.00	0.00	100.00	ON160849
TU-33	*Aspergillus arcoverdensis*	100.00	0.00	100.00	ON160850
TU-35	*Aspergillus niger*	100.00	0.00	100.00	ON160851
TU-36	*Aspergillus europaeus*	100.00	0.00	100.00	ON160852

**Figure 3:**
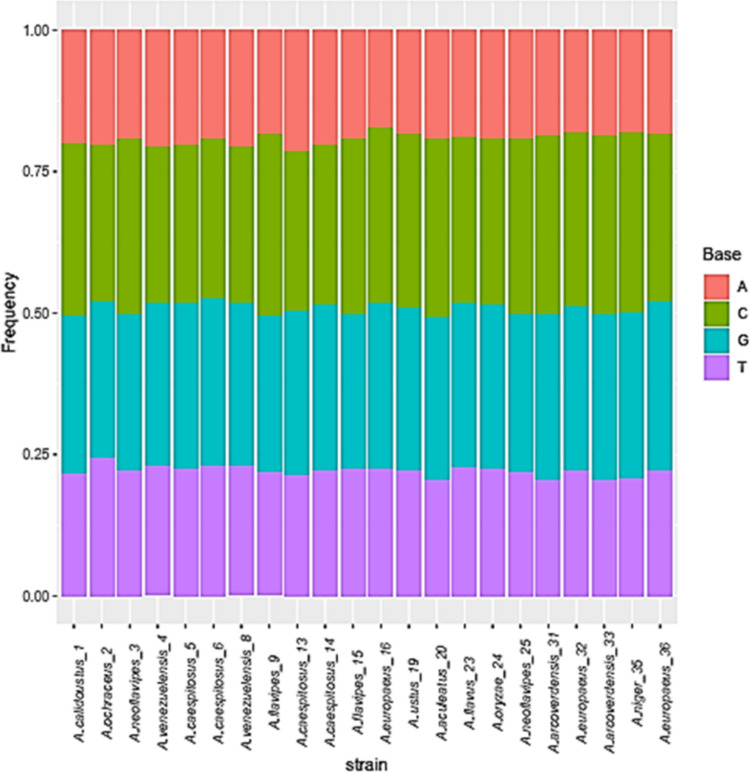
Base frequencies of 22 *Aspergillus* ITS isolates.

**Figure 4:**
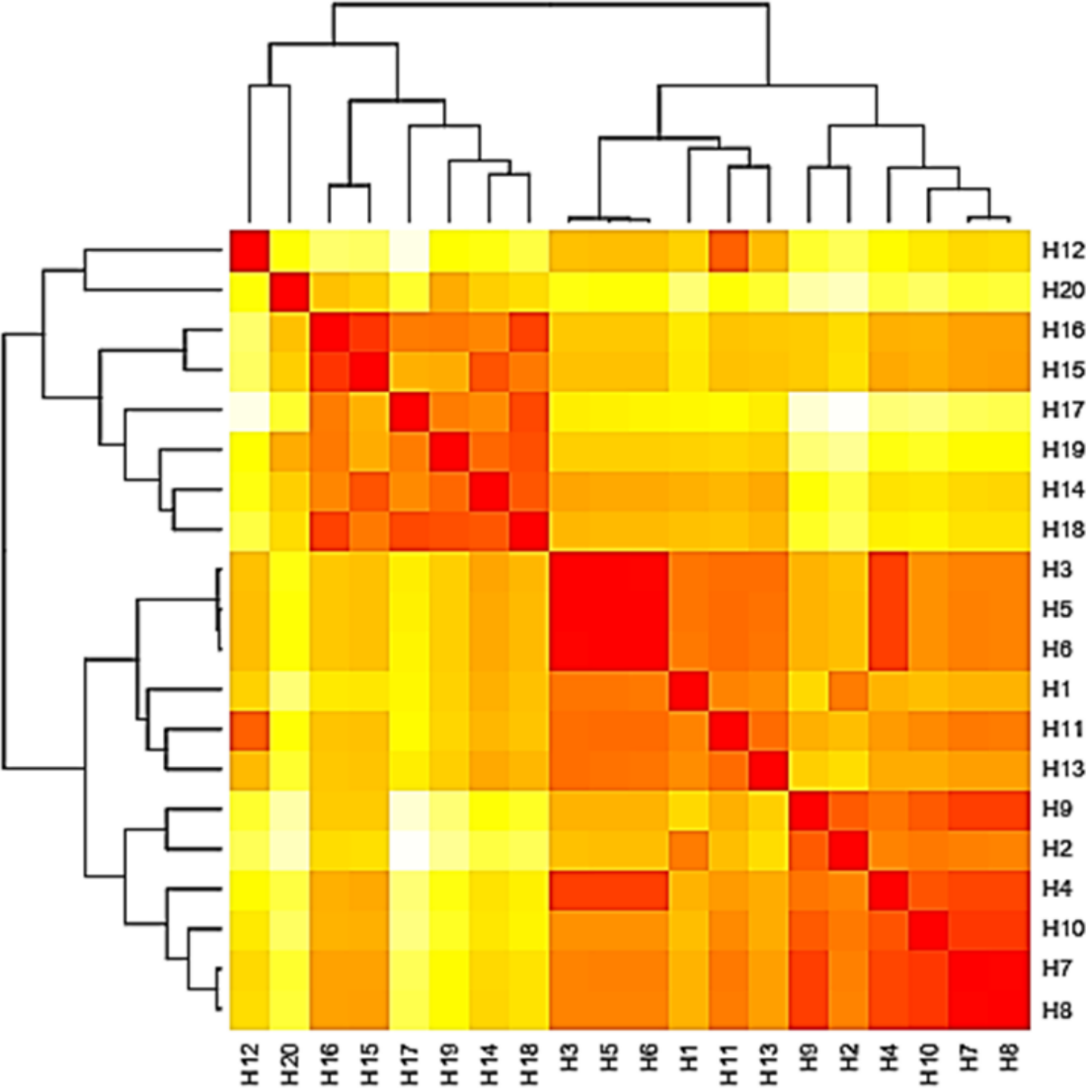
Heat map based on the number of nucleotide differences between the haplotypes. Each branch of the phylogenetic tree represents the corresponding haplotype in the matrix. The close relationships were defined by a darker color and distant relationships by a lighter color.

**Figure 5:**
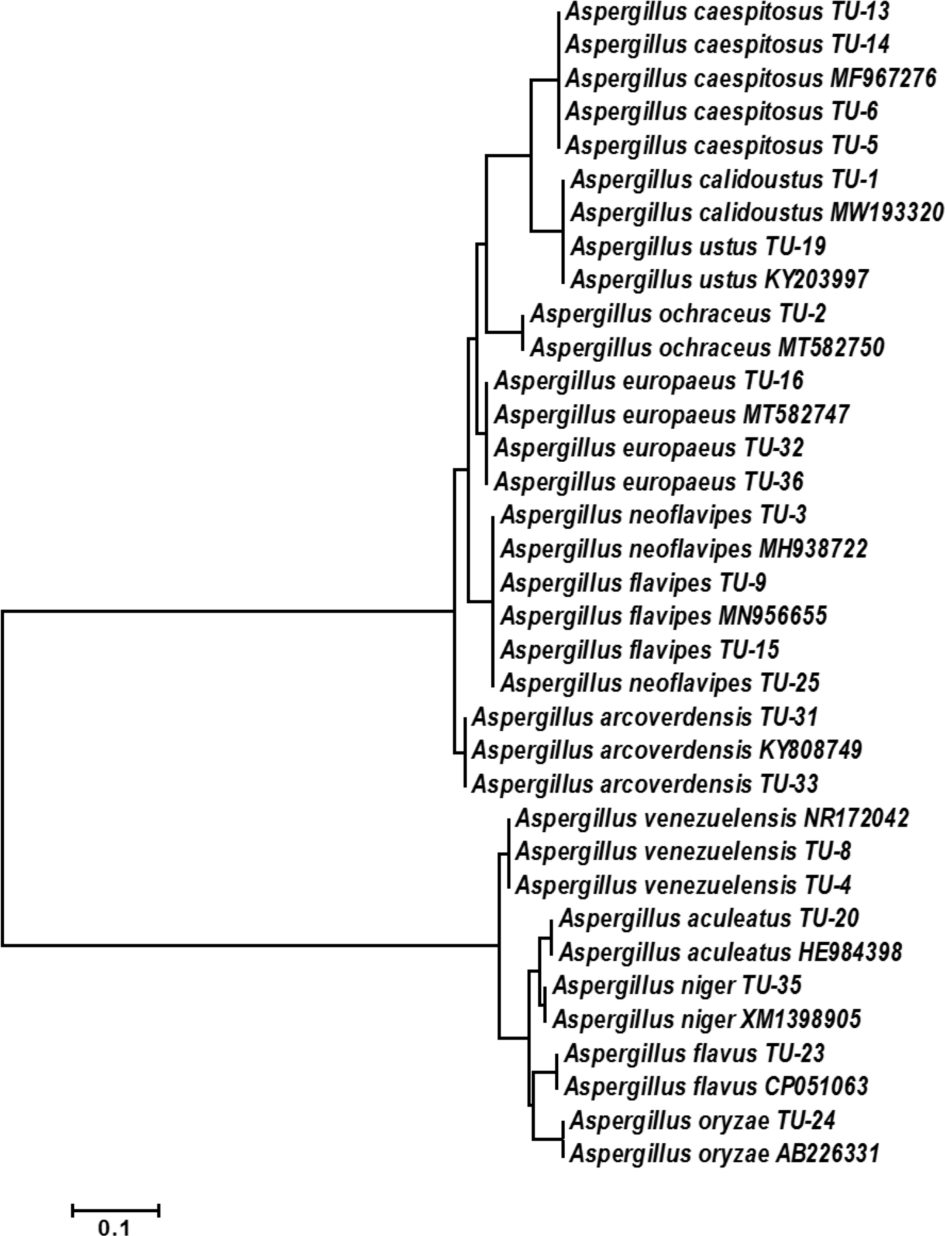
Phylogenetic tree and diversity of the 5.8S-ITS region with different *Aspergillus* species compared with reference *Aspergillus* strains.

The base frequencies of all ITS sequenced isolates were shown in Fig. 3. A heat map using phylogenetic trees from the haplotype sequences matrix was shown in Fig. 4. The phylogenetic tree based on the 5.8S-ITS region sequence with 1,000 bootstrap repeats showed that the phylogeny tree contained two main clusters (Fig. 5 and Fig. S1). The first main cluster contained five sub-cluster with a bootstrap value of 99%. The first one contained *Aspergillus europaeus* TU-16, 32 and 36 with 99–100% similarity to *A. europaeus* MT582747 from NCBI GenBank. The second sub-cluster contained *A. flavus* TU-23 with 100% similarity to *A. flavus* MT635198, it contained also *A. oryzae* TU-24 with high similarity to *A. oryzae* MW331693. Moreover, the third sub-cluster contained *A. arcoverdensis* TU-31 and TU-33 which were homologous with *A. arcoverdensis* KY808749. The fourth sub-cluster contained *A. aculeatus, A. niger, A. aculeatus* TU-20 which were homologous with *A. aculeatus* MN795742 and *A. niger* TU-35 which was homologous with *A. niger* MT541880. The final sub-cluster contained *A. neoflavipes* and *A. flavipes*. *A. flavipes* TU-9 and TU-15 were homologous with *A. flavipes* MN956655, while *A. neoflavipes* TU-3 and TU-25 were homologous with *A. neoflavipes* MH938722. On the other hand, the second main cluster contained two subclusters. The first one contained *A. ochraceus* TU-2 with 100% similarity to *A. ochraceus* MT582750. The second sub-cluster contained three groups, the first one contained *A. calidoustus* TU-1 which was homologous with *A. calidoustus* MW193320 and *A. ustus* TU-19 which were homologous with KY203997. The second one contained *A. venezuelensis* TU-4 and TU-8 which were homologous with *A. venezuelensis* NR172042. The last group contained *A. caespitosus* TU-5, TU-6, TU-13 and TU-14 which were homologous with *A. caespitosus* MF967276 (Fig. 3).

### Molecular identification of *Aspergillus* species based on TEF-1 *α* DNA gene

To evaluate the applicability of TEF-1 *α* DNA sequences for the differentiation and phylogenetic study of *Aspergillus* species, partial TEF-1 *α* genes were examined for 22 strains of *Aspergillus* (Table 5). Using the primer pair TEF-1 *α* F and TEF-1 *α* R, we were able to successfully amplify a single PCR fragment for all of the *Aspergillus* strains included in the study, and this was followed by analysis of the DNA sequences and a nucleotide-based phylogenetic analysis. Multiple alignment of sequences pertaining to TEF-1 *α* indicated a mean similarity of 92.6% between the species. A BLAST search was run on the acquired sequences to determine the identities of the isolates, and the results were deposited in the GenBank database (accession numbers ON456514 –ON456535) (Table 5).

**Table 5:** The NCBI BLAST query of *TEF* gene sequences for *Aspergillus* species isolated from invertebrates in Taif, Saudi Arabia.

Isolates	Species	Query coverage %	E value	Ident %	Accession number
TU-1	*Aspergillus calidoustus*	100	0.00	100	ON456514
TU-2	*Aspergillus ochraceus*	100	0.00	99	ON456515
TU-3	*Aspergillus neoflavipes*	99	0.00	98	ON456516
TU-4	*Aspergillus venezuelensis*	100	0.00	99	ON456517
TU-5	*Aspergillus caespitosus*	100	0.00	99	ON456518
TU-6	*Aspergillus caespitosus*	99	0.00	99	ON456519
TU-8	*Aspergillus venezuelensis*	99	0.00	98	ON456520
TU-9	*Aspergillus flavipes*	99	0.00	98	ON456521
TU-13	*Aspergillus caespitosus*	99	0.00	98	ON456522
TU-14	*Aspergillus caespitosus*	99	0.00	99	ON456523
TU-15	*Aspergillus flavipes*	99	0.00	99	ON456524
TU-16	*Aspergillus europaeus*	99	0.00	99	ON456525
TU-19	*Aspergillus ustus*	100	0.00	100	ON456526
TU-20	*Aspergillus aculeatus*	100	0.00	100	ON456527
TU-23	*Aspergillus oryzae*	99	0.00	98	ON456528
TU-24	*Aspergillus flavus*	100	0.00	99	ON456529
TU-25	*Aspergillus neoflavipes*	100	0.00	99	ON456530
TU-31	*Aspergillus arcoverdensis*	100	0.00	100	ON456531
TU-32	*Aspergillus europaeus*	100	0.00	100	ON456532
TU-33	*Aspergillus arcoverdensis*	99	0.00	100	ON456533
TU-35	*Aspergillus niger*	99	0.00	100	ON456534
TU-36	*Aspergillus europaeus*	99	0.00	100	ON456535

**Figure 6:**
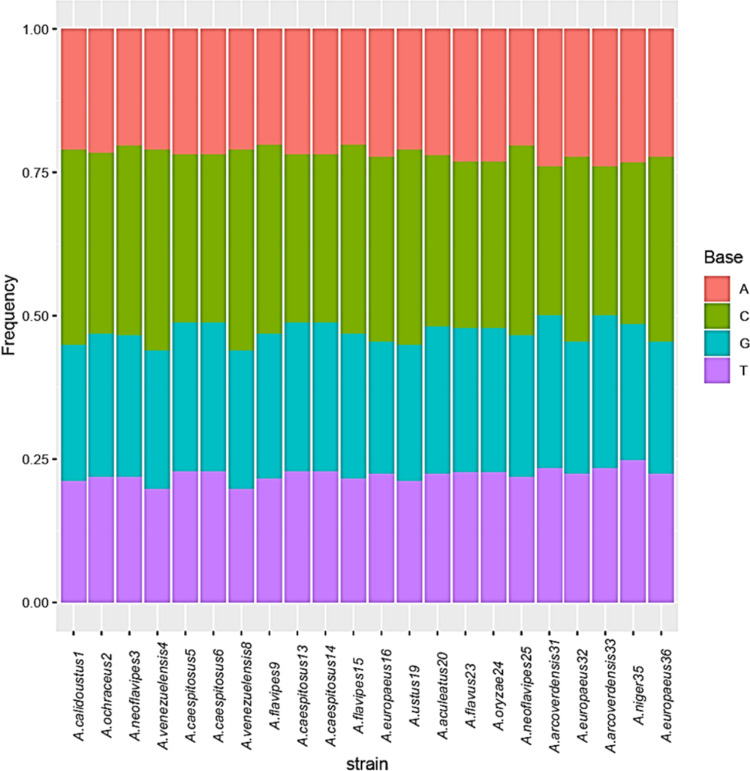
Base frequencies of 22 TEF *Aspergillus* isolates.

**Figure 7:**
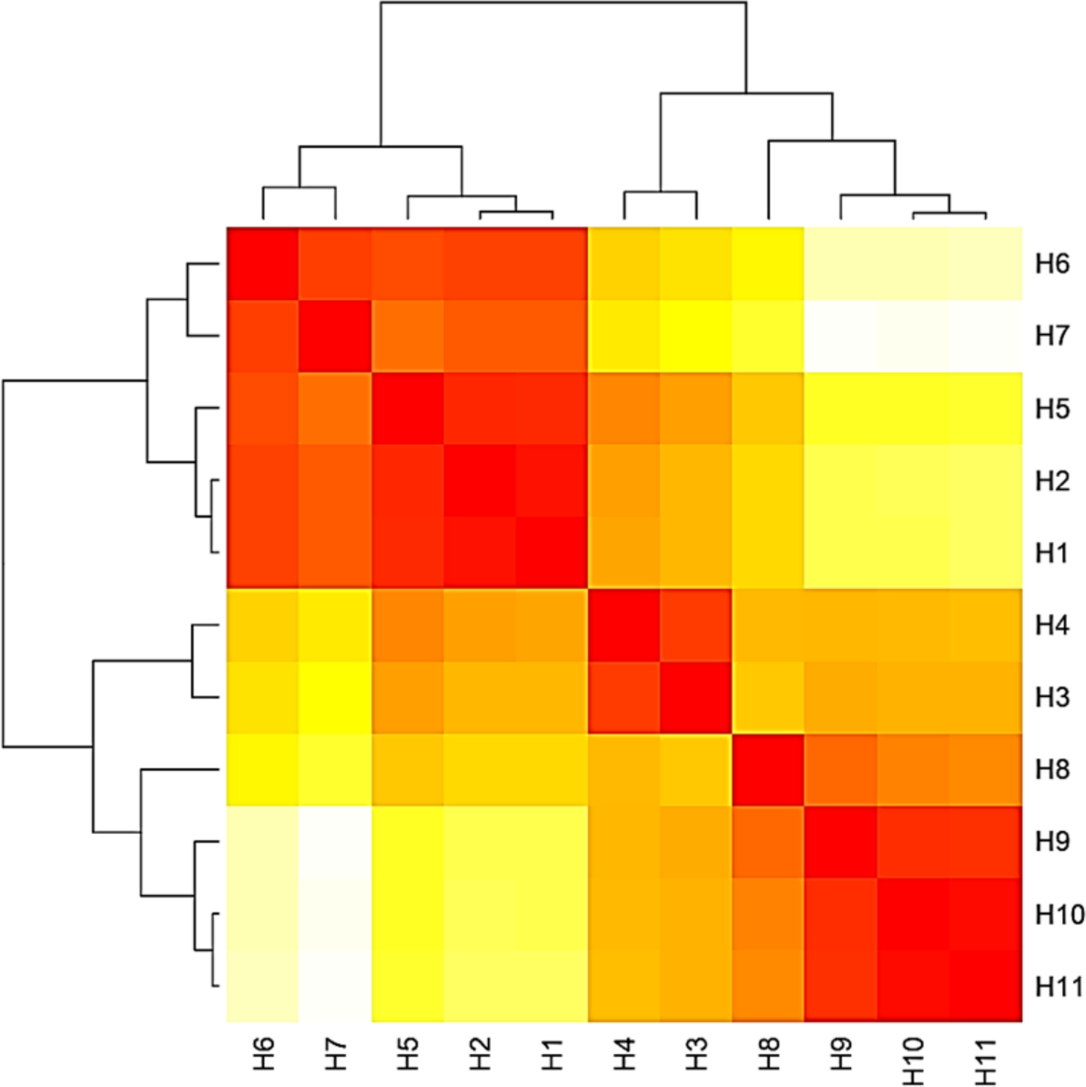
Heat map based on the number of nucleotide differences between the haplotypes. Each branch of the phylogenetic tree represents the corresponding haplotype in the matrix. The close relationships were defined by the darker color and distant relationship by the lighter color.

**Figure 8:**
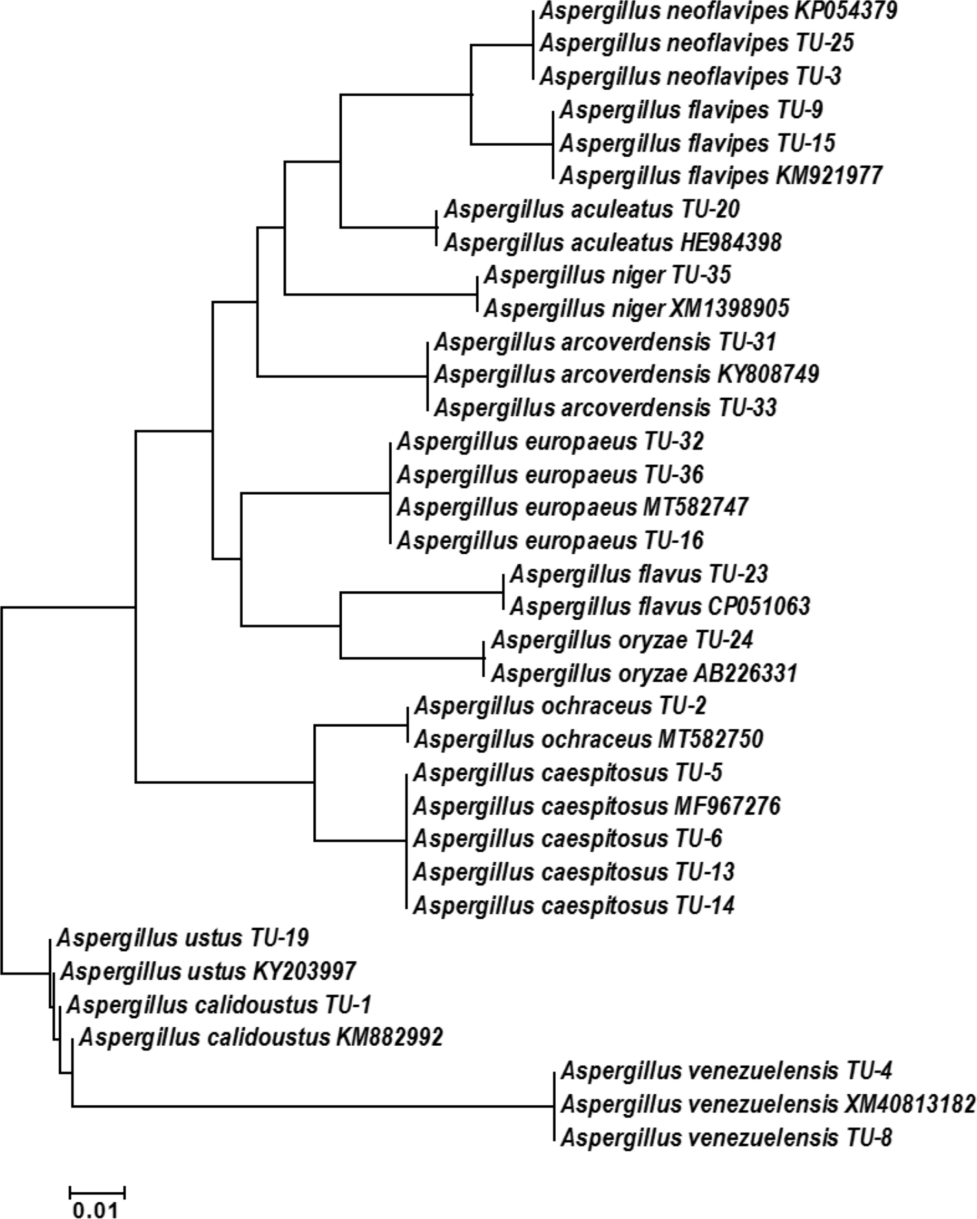
Phylogenetic tree and diversity of the TEF-1 *α* gene with different *Aspergillus* species compared with reference *Aspergillus* strains. The phylogenetic tree was generated using parsimony neighbor-joining and maximum likelihood analysis.

The base frequencies of all TEF-1 *α* sequenced isolates were explained (Fig. 6). From the matrix of haplotype sequences, phylogenetic trees were generated and used to create a heat map (Fig. 7). The phylogenetic tree based on the TEF-1 *α* DNA sequence with 1,000 bootstrap repeats showed that the phylogeny tree contained two main clusters (Fig. 8 and Fig. S2). The first main cluster contained eight sub-cluster with a bootstrap value of 99–100%. The first sub-cluster contained *A. neoflavipes* and *A. flavipes*. *A. flavipes* TU-9 and TU-15 were found to be homologs with *A. flavipes* MN956655, while *A. neoflavipes* TU-3 and TU-25 were found to be homologs with *A. neoflavipes* KM921977. The second sub-cluster contained *A. aculeatus* TU-20 which was homologs with *A. aculeatus* HE984398. The third one contained *A. ochraceus* TU-2 with 100% similarity to *A. ochraceus* MT582750. The fourth sub-cluster contained *A. arcoverdensis* TU-31 and TU-33 with 100% similarity to *A. arcoverdensis* KY808749. The fifth sub-cluster contained *A. niger* TU-35 which was found to be homologs with *A. niger* XM1398905. The sixth sub-cluster contained *A. europaeus* TU-16, 32 and 36 with 100% similarity to *A. europaeus* MT582747. The seventh subcluster contained *A. flavus* TU-23 with 100% similarity to *A. flavus* CP051063, it contained also *A. oryzae* TU-24 with high similarity to *A. oryzae* AB226331. The final sub-cluster contained *A. caespitosus* TU-5, TU-6, TU-13 and TU-14 which was found to be homologs with *A. caespitosus* MF967276. On the other hand, the second main cluster contained two sub-clusters. The first one contained *A. calidoustus* TU-1 which was found to be homologs with *A. calidoustus* KM882992 and *A. ustus* TU-19 which was found to be homologs with KY203997. Finally, the second sub-cluster contained *A. venezuelensis* TU-4 and TU-8 which were homologs with *A. venezuelensis* XM813182.

### ISSR-PCR analysis

**Figure 9:**
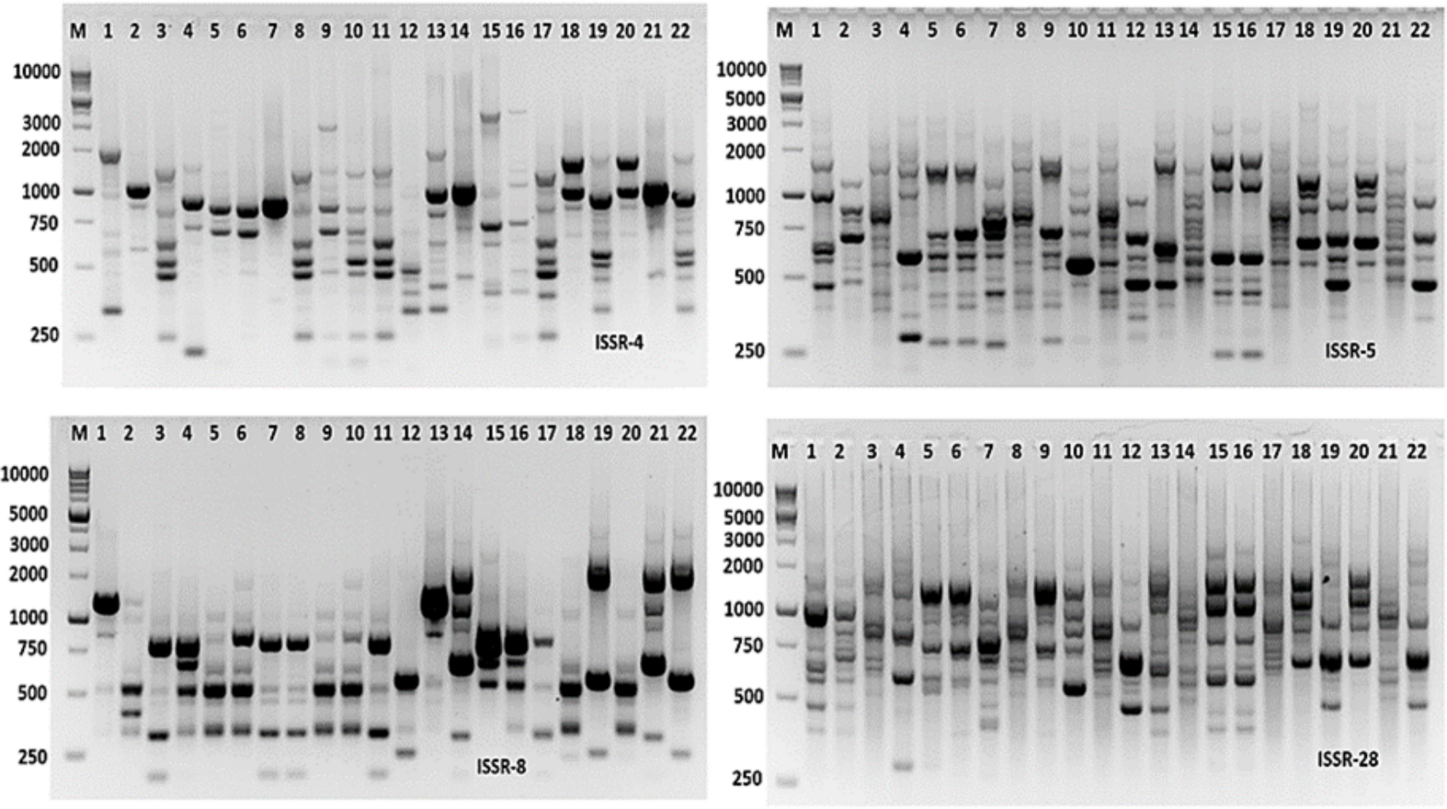
ISSR-PCR profile of the 22 *Aspergillus* isolates generated with ISSR primers. M is 100 bp DNA ladder.

Molecular markers are effective tools for molecular characterization and correlation estimation through DNA fingerprinting. *Aspergillus* species were molecular characterized using ISSR-PCR markers. The ISSR-PCR results were summarized in Table 6 and shown in Fig. 9. The polymorphic and monomorphic bands were produced from the PCR amplification. About 92 bands resulted from the five ISSR-PCR primers. Out of them, 32 bands were monomorphic with a monomorphism average of 34.8%, while 60 bands were polymorphic bands with a polymorphism average of 65.2%. The number of total bands varied from 14 to 22 bands, with primers ISSR-8 and ISSR-5, respectively. The band’s size ranging from 220 to 2,800 bp with primer ISSR-5. The highest polymorphism among *Aspergillus* species was revealed by ISSR-4 primer (76.2%), followed by ISSR-5 primer (68.2%). However, the lowest polymorphism was 57.82% resulting from the application of ISSR-28 primer.

### Genetic distances and the cluster dendrogram

**Figure 10:**
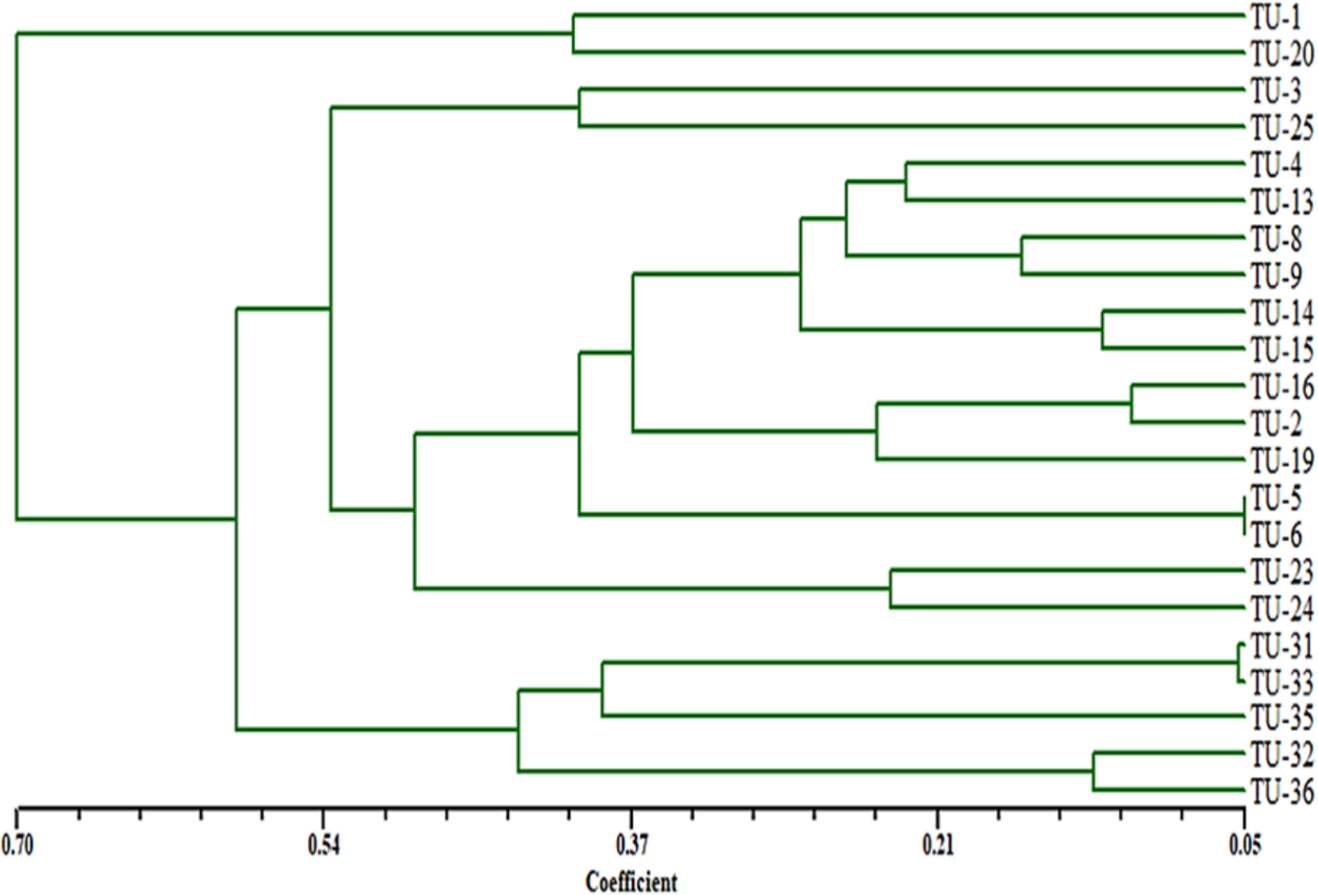
Dendrogram of genetic similarity and inter and intra-species diversity of the *Aspergillus* isolates.

A total of 92 fragments from all ISSR-PCR analyses were enough to determine genetic similarities and design the phylogenetic tree for these *Aspergillus* isolates. According to a dendrogram constructed using UPGMA based on Jaccard’s similarity coefficients dependent on genetic similarity, inter and intra-species diversity ranged from 0.05 to 0.70 (Fig. 10), the *Aspergillus* isolates were grouped into two different clusters with about 65% genetic similarity. The first cluster contained only *Aspergillus* TU-1 and TU-20. While, the second cluster contained most *Aspergillus* isolates. The second cluster contained two sub-cluster, the first one contained *Aspergillus* TU-3 and TU-25 in the same group, *Aspergillus* TU-4, TU-13, TU-8, TU-9, TU-14, TU-15, TU-16, TU-2, TU-19, TU-5, TU-6, TU-23 and TU-24 were found in the second group. Moreover, the second sub-cluster contained *Aspergillus* TU-31, TU-32, TU-33, and TU-35 and TU-36. The dendrogram showed the highest genetic similarity between *Aspergillus* TU-5 and TU-6, while the lowest relationship was between *Aspergillus* TU-1 and TU-36. The dendrogram constructed using UPGMA based on Jaccard’s similarity coefficients (Fig. 6) indicated that *Aspergillus* TU-2 and TU-16 were in the same sub-cluster and appeared more similar to each other than *Aspergillus* TU-19.

### Detection of aflatoxin and ochratoxin-producing genes

**Figure 11:**
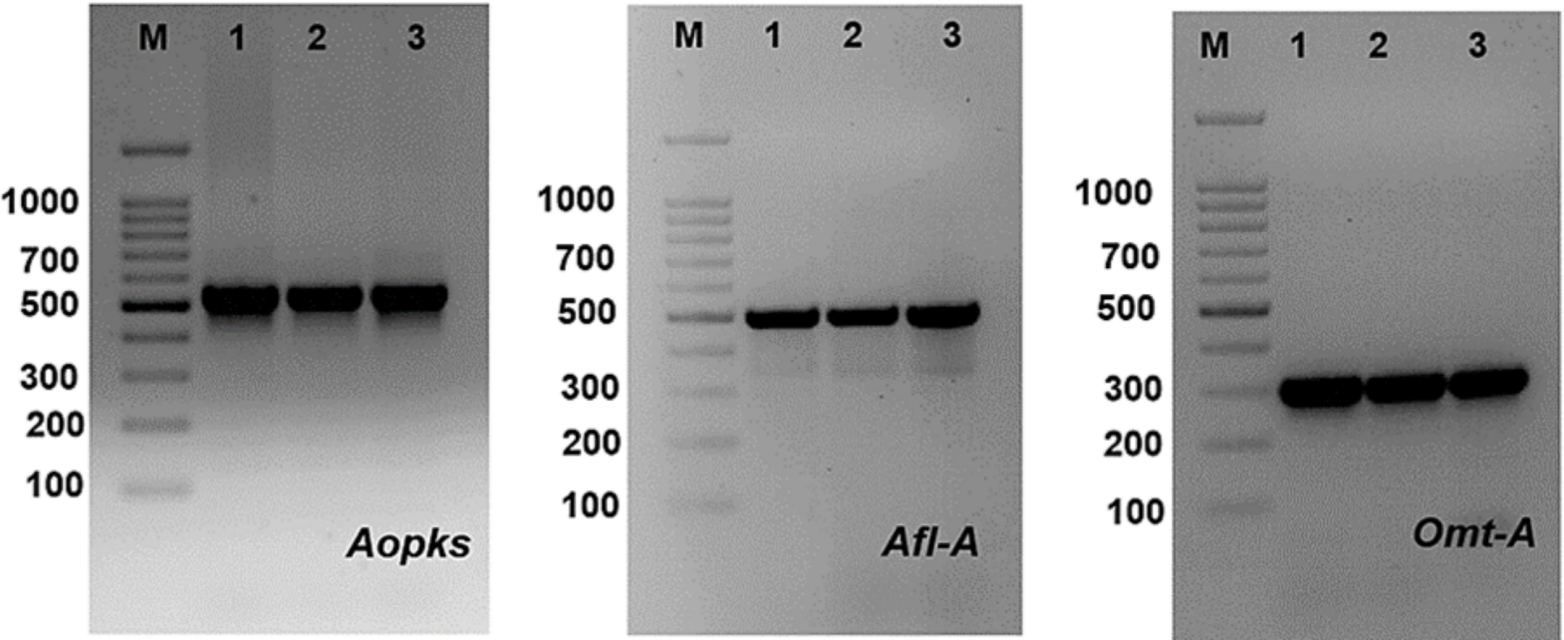
PCR amplification of some mycotoxin genes in studied *Aspergillus* species, where *Aopks, Afl-A* and *Omt-A* genes molecular weight are (549, 497 and 300 bp), respectively.

PCR technique using four sets of primers was used to discover different genes involved in the biosynthetic pathways of mycotoxin (aflatoxin and ochratoxin). Bands of the fragments of *afl-A*, *omt-A* and *Aopks* genes can be visualized at 497, 300 and 549 bp, respectively (Table 7, Fig. 11 and Fig. S3). Aflatoxigenic strains (*Aspergillus ochraceus* TU-2, *Aspergillus neoflavipes* TU-3, *Aspergillus flavipes* TU-9, *Aspergillus caespitosus* TU-13, *Aspergillus caespitosus* TU-14, *Aspergillus flavipes* TU-15, *Aspergillus ustus* TU-19, *Aspergillus aculeatus* TU-20, *Aspergillus oryzae* TU-23, *Aspergillus flavus* TU-24, *Aspergillus neoflavipes* TU-25, *Aspergillus arcoverdensis* TU-31, *Aspergillus europaeus* TU-32, *Aspergillus arcoverdensis* TU-33, and *Aspergillus europaeus* TU-36) showed 497 bp DNA fragments with *afl-A* primer. Moreover, Aflatoxigenic strains (*Aspergillus oryzae* TU-23, *Aspergillus flavus* TU-24, *Aspergillus europaeus* TU-32 and *Aspergillus europaeus* TU-36) showed 300 bp DNA fragments that corresponded to the complete aflatoxin B1 producing gene. In addition, *Aspergillus arcoverdensis* TU-31, *Aspergillus arcoverdensis* TU-33 and *Aspergillus niger* TU-35 showed 549 bp DNA fragments with *Aopks* primer. On the other hand, some strains have the phosphate solubilization gene, glucose dehydrogenase (Gdh), such as *Aspergillus flavipes* TU-15, *Aspergillus oryzae* TU-23, *Aspergillus flavus* TU-24, *Aspergillus neoflavipes* TU-25 and *Aspergillus europaeus* TU-32.

**Table 6:** Polymorphism level detected by the five ISSR primers that have been used for ISSR-PCR analysis.

Primers	Total bands	Monomorphic bands	Polymorphic bands	Monomorphism (%)	Polymorphism (%)
ISSR-4	21	5	16	23.80	76.20
ISSR-5	22	7	15	31.80	68.20
ISSR-8	14	6	8	42.85	58.15
ISSR-18	18	7	11	38.89	61.11
ISSR-28	17	7	10	41.18	57.82
Total	92	32	60		

**Table 7:** The NCBI BLAST query of ITS region sequences for *Aspergillus* species isolated from invertebrates in Taif, Saudi Arabia.

Isolates	Species	Gdh gene	AflA gene	OmtA gene	Aopks gene
TU-1	*Aspergillus calidoustus*	–	–	–	–
TU-2	*Aspergillus ochraceus*	–	+	–	–
TU-3	*Aspergillus neoflavipes*	–	+	–	–
TU-4	*Aspergillus venezuelensis*	–	–	–	–
TU-5	*Aspergillus caespitosus*	–	–	–	–
TU-6	*Aspergillus caespitosus*	–	–	–	–
TU-8	*Aspergillus venezuelensis*	–	–	–	–
TU-9	*Aspergillus flavipes*	–	+	–	–
TU-13	*Aspergillus caespitosus*	–	+	–	–
TU-14	*Aspergillus caespitosus*	–	+	–	–
TU-15	*Aspergillus flavipes*	+	+	–	–
TU-16	*Aspergillus europaeus*	–	–	–	–
TU-19	*Aspergillus ustus*	–	+	–	–
TU-20	*Aspergillus aculeatus*	–	+	–	–
TU-23	*Aspergillus oryzae*	+	+	+	–
TU-24	*Aspergillus flavus*	+	+	+	–
TU-25	*Aspergillus neoflavipes*	+	+	–	–
TU-31	*Aspergillus arcoverdensis*	–	+	–	+
TU-32	*Aspergillus europaeus*	+	+	+	–
TU-33	*Aspergillus arcoverdensis*	–	+	–	+
TU-35	*Aspergillus niger*	–	–	–	+
TU-36	*Aspergillus europaeus*	–	+	+	–

## Discussion

The long-term existence of species depends on genetic diversity, which is also crucial to their protection (Frankham, 2012). In addition, genetic diversity is crucial for how populations react to environmental variables (Hoelzel, Bruford & Fleischer, 2019). Consequently, understanding the genetic diversity of populations is crucial for the preservation and appropriate use of genetic resources, as well as their underlying individual and subpopulation components (Pazouki et al., 2015). Species of the genus *Aspergillus* are widely dispersed everywhere in the environment (Krishnan, Manavathu & Chandrasekar, 2009; Mazrou et al., 2020). The genus is divided into seven subgenera, subdivided into a number of sections, each containing a few to several closely related species (Nouripour-Sisakht et al., 2017). The morphological characteristics of the isolated *Aspergillus* species were investigated. The colony of *A. ochraceus* TU2 was yellow-gold on Czapek Agar (CZA) medium. Spherical small conidia and smooth finely roughened (Raper & Fennell, 1965; Nyongesa, Okoth & Ayugi, 2015). While, *A. europaeus* was yellowish grey, velutinous to floccose and Sclerotia and Hülle cells were absent (Nguyen et al., 2020). The colony of *A. flavus* was granular, floccose, yellowish green and cleistothecia was present (Raper & Fennell, 1965; Jernejc & Cimerman, 2001; Gautam & Bhadauria, 2012; Zulkifli & Zakaria, 2017; Dewi, 2018). Colonies of *A. flavipes* and *A. neoflavipes* were floccose to cottony. Hülle cells were absent and, ascomata (Hubka et al., 2015). On the other hand, the colony of *Aspergillus arcoverdensis* was white to orange, white, floccose, and cleistothecia was not present (Samson et al., 2014; Matsuzawa et al., 2015). Conidia of *A. caespitosus* and *A. venezuelensis* were globose, globose to subglobose. Ascomata, ascospores, globose and Hülle cells were present (Samson et al., 2014; Chen et al., 2016). On the other side, colonies of *Aspergillus aculeatus* and *A. niger* were dark brown/gray tones, dark brown to black, velvety, granular, and pale to yellow. Fruiting bodies were absent (Raper & Fennell, 1965; Jernejc & Cimerman, 2001; Silva et al., 2011; Gautam & Bhadauria, 2012; Samson et al., 2014; Nyongesa, Okoth & Ayugi, 2015; Dewi, 2018). While, colonies of *A. calidoustus* and *A. ustus* were floccose, blond/greyish yellow, and greyish brown to dark brown. Conidiophore was brown and smooth (Raper & Fennell, 1965; Houbraken et al., 2007).

*Aspergillus* species’ taxonomy and evolutionary relationships are based on the morphological characteristics, extrapolated data and partial DNA sequences of different genetic targets (Varga, Frisvad & Samson, 2011). However, lack of pigment or poor sporulation, inter-specific similarities and intra-specific variability, and variation in growth requirements for some isolates may influence the outcome of morphological identification (Rodrigues et al., 2009). Therefore, molecular methods are necessary for distinguishing and/or (re-) classifying similar and complex *Aspergillus* taxa, as well as for the discovery of new species. An international *Aspergillus* working group has recommended the use of molecular identification based on the ITS region for subgenus/section-level identification (Hu et al., 2021). However, comparative sequence-based identification using the ITS1 region does not always enable discrimination between closely related species (Samson et al., 2014), which might be due to inadequate sequence variability or issues with the reliability of the ITS sequences deposited in reference databases (Nilsson et al., 2006). Some housekeeping genes, such as BT2, calmodulin and rodlet A, have been confirmed as good genetic indicators for identifying species within different sections, such as Fumigati, Usti, Nigri and Terrei (Houbraken et al., 2020), but they are not able to differentiate, for example, species that are phylogenetically close to *A. parasiticus* (Vesth et al., 2018), and therefore sequence analyses of other loci are required for accurate identification.

Owing to polymorphism and DNA sequence length differences in introns, intron-rich protein-coding genes such as *β*-tubulin, calmodulin, actin and TEF-1 *α* are recommended for the discrimination of fungal species (Sklenář et al., 2020). Hence, the identical size (700 nt) and high homology (92.6%) observed among all the strains reflected the lack of introns in the TEF-1a gene region studied here. Phylogenetic analysis of the TEF-1 *α* genes showed a clade consisting of *A. flavus*, *A. oryzae*, *A. ochraceus* and *A. tamari* (*Flavi* section), supported by a bootstrap value of 97%, next to the members of *A. avenaceus* and *A. alliaceus*, as a separate group with a bootstrap value of 100% REFF. Pairwise comparison of the DNA sequences of both the ITS and TEF-1 *α* genes in this study showed a single SNP difference between *A. flavus* and *A. oryzae*. Regarding the evolutionary origins of *A. oryzae* and *A. flavus*, based on the region neighboring the cyclopiazonic acid biosynthesis gene cluster, Chen et al. (2016) suggested that *A. oryzae* most likely descended from an ancestor that was the ancestor of *A. minisclerotigenes* or *A. parvisclerotigenus*, producing both B- and G-type aflatoxins, while *A. flavus* descended from an ancestor of *A. parasiticus*. Although several lines of evidence show that *A. oryzae* is a morphological variant of *A. flavus*, it was suggested that these taxa should be retained as separate species because of the regular confusion that conspecificity might generate in the food industry (Varga, Frisvad & Samson, 2011; Rodrigues et al., 2009).

Differentiation of some species of black aspergilli, such as *A. niger* and *A. tubingensis*, which are common in both clinical and environmental settings, remains difficult (Criseo, Bagnara & Bisignano, 2001). The taxa can be differentiated by DNA sequences reflecting the cytochrome b (Schmidt-Heydt et al., 2009), ITS (Abdel-Hadi, Carter & Magan, 2011) and *β*-tubulin (Mutegi et al., 2012) genes. In this study, a sequence difference count matrix based on nt pairwise comparison of the TEF-1 *α* gene with a similarity of 81.8% provided evidence that this locus is more valuable than ITS (97.9% similarity) for species discrimination of these two closely related species.

The phylogenetic tree of the TEF-1 *α* genes revealed a cluster consisting of *Fumigati* and *Clavati* sections. Closely related species, *A. fumigatus*, *A. fischeri* and *A. quadricinecta* (section *Fumigati*), formed well-supported clades in the TEF-1 *α* and BenA gene analyses, with bootstrap values of 98 and 100%, respectively. The phylogenetic tree derived from TEF-1a indicated that *A. clavatus*, *A. clavatonanicus* and *A. nutans* (section *Clavati*) form a sister group with section Fumigati, with a bootstrap value of 76%, and the phylogenetic analysis of partial DNA sequences of BT2 genes provided a bootstrap value of 100%.

Many researchers have investigated the function of fungi in the contamination and generation of aflatoxin in different crops (El-Shanshoury et al., 2014). Studying mycotoxigenic fungus’s molecular genetics and metabolism is an intriguing approach to reducing crop contamination with mycotoxin (El-Kady & Youssef, 1993). The study of the genetic variations between both aflatoxigenic and non-aflatoxigenic strains is of interest. It is estimated that between 300 and 400 different mycotoxins exist, but the ones produced by *Aspergillus* have received the most attention due to their potential impact on human, animal, and plant health. *Aspergillus* species are responsible for the production of numerous severe and fatal biotoxins. These include aflatoxins (AFTs), patulin (PAT), ochratoxins (OTA), aflatrem (AT), citrinin (CIT), cyclopiazonic acid (CPA), secalonic acids (SA), sterigmatocystin (ST), terrein (TR), and gliotoxin (GT) (Ráduly et al., 2020). This knowledge has been utilized for controlling the production of aflatoxins. This study utilized traditional PCR techniques to explore the presence of mycotoxin genes in different *Aspergillus* isolates which should be useful to understand mycotoxins contamination in the soil.

## Conclusion

Herein, various genera of fungi were isolated from soil invertebrates (millipedes, *Armadillidium vulgare* and *Porcellio laevis*) in Taif Governorate, Saudi Arabia. The isolates were identified based on the morphological characteristics and molecular analysis of ITS and TEF DNA sequences. *Aspergillus* spp. were found to be the most common in the samples. ISSR-PCR markers were used to study the genetic diversity of *Aspergillus* isolates. Detection and identification of mycotoxin genes in *Aspergillus* isolates were revealed.

##  Supplemental Information

10.7717/peerj.15035/supp-1 Supplemental Information 1 Phylogenetic tree (with bootstrap) and diversity of the 5.8S-ITS region with different *Aspergillus* species compared with reference *Aspergillus* strains

10.7717/peerj.15035/supp-2 Supplemental Information 2 Phylogenetic tree (with bootstrap) and diversity of the TEF-1 *α* gene with different Aspergillus species compared with reference Aspergillus strains

10.7717/peerj.15035/supp-3 Supplemental Information 3 PCR amplification of some mycotoxin genes in studied *Aspergillus* species, where A* is Gdh gene, B is Aopks,* C is Omt-A* and D is Afl-A* gene

10.7717/peerj.15035/supp-4 Supplemental Information 4 Sequence of ITS of Aspergillus isolates

10.7717/peerj.15035/supp-5 Supplemental Information 5 Sequence of Tef gene of Aspergillus isolates

## Additional Information and Declarations

### DNA Deposition

The following information was supplied regarding the deposition of DNA sequences: The sequences are available at GenBank: ON160831–ON160840.

### Data Availability

The following information was supplied regarding data availability: The raw data is available in the Supplementary Files.
