# Supplementary figures and images for: Identification and biodiversity patterns of Aspergillus species isolated from some soil invertebrates at high altitude using morphological characteristics and phylogenetic analyses
Source: PeerJ. 2023 Mar 28;11:e15035. doi: 10.7717/peerj.15035 (PMC10075209; doi:10.7717/peerj.15035)

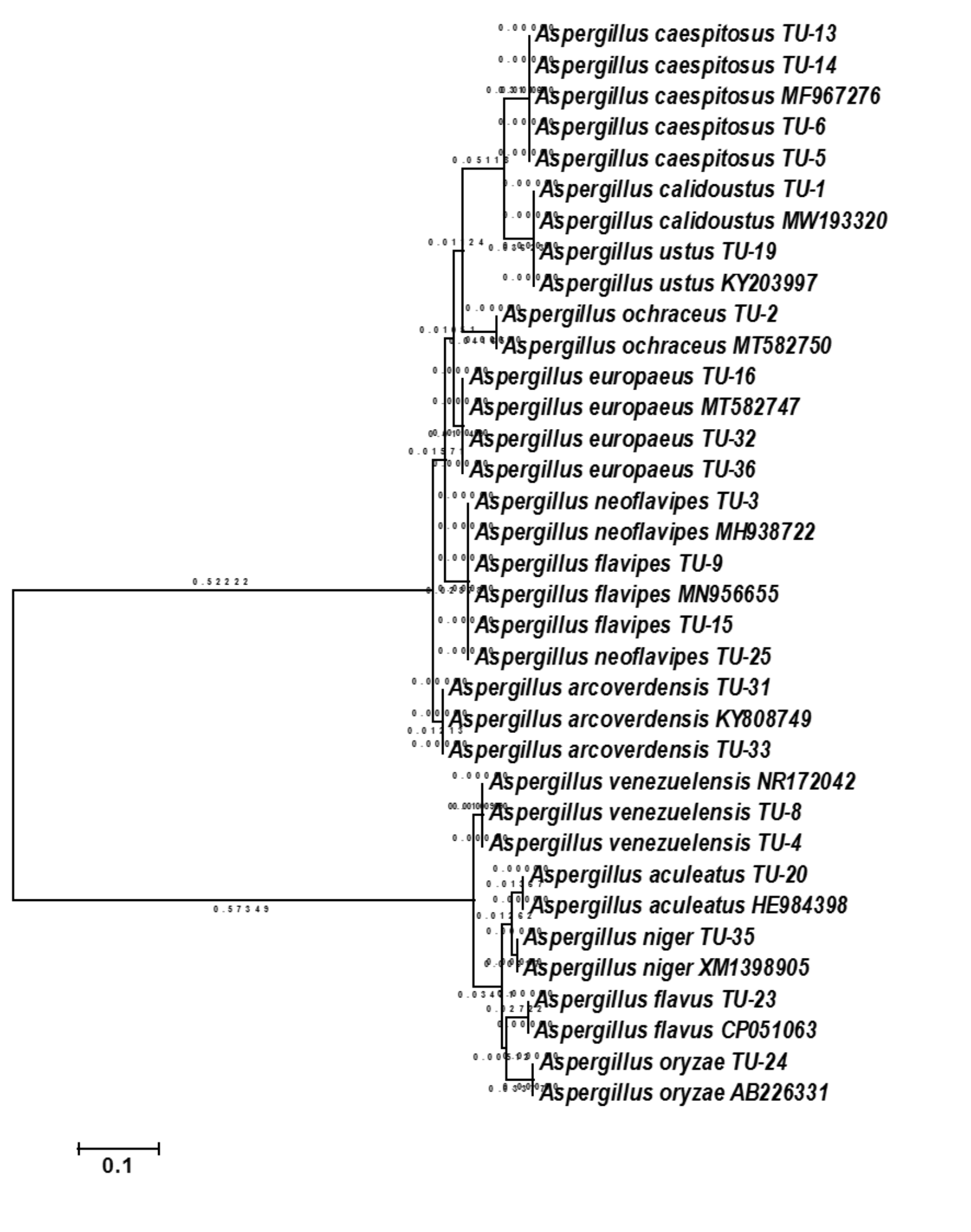

Supplement: Supplemental Information 1 [file peerj-11-15035-s001.png]

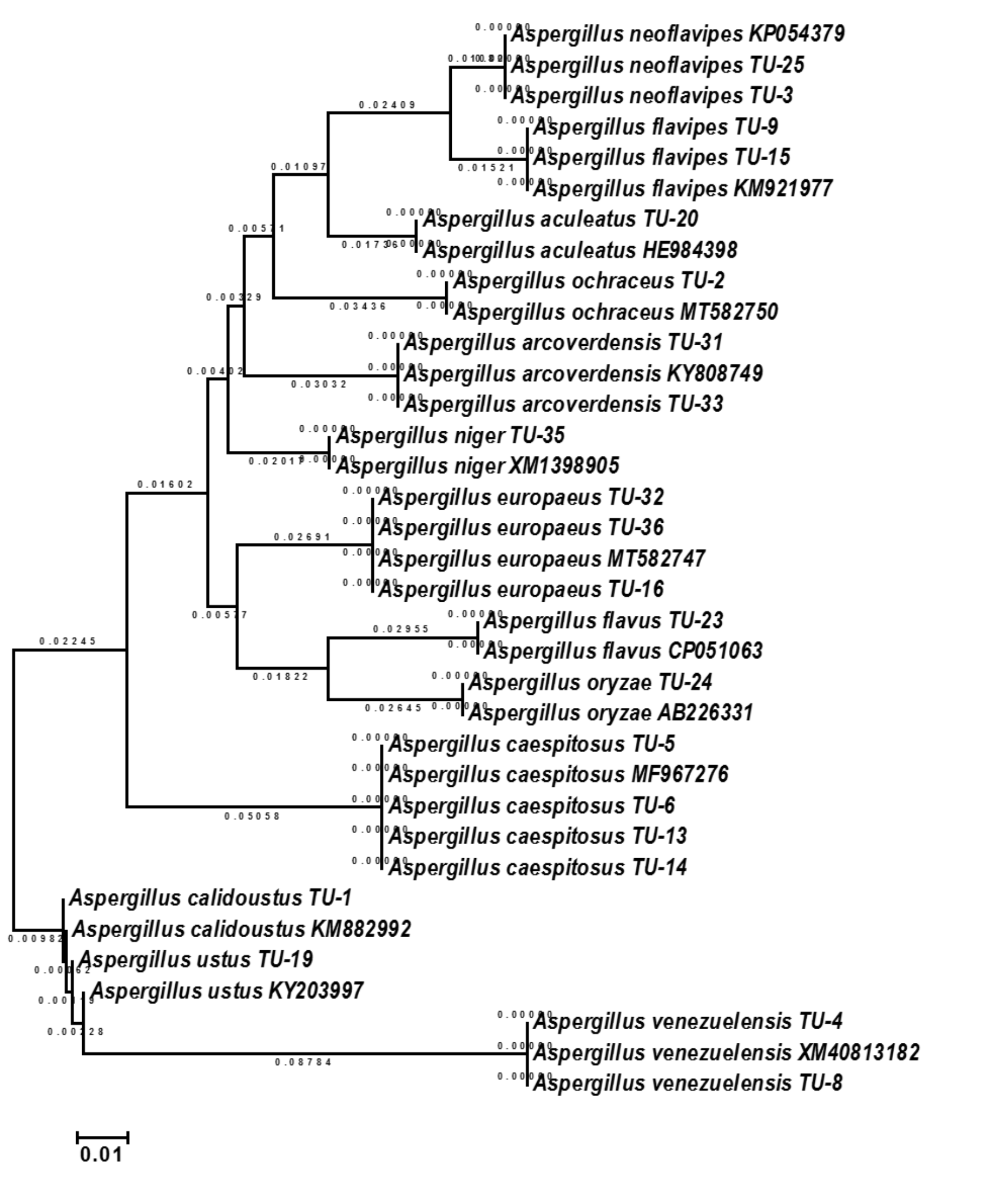

Supplement: Supplemental Information 2 [file peerj-11-15035-s002.png]

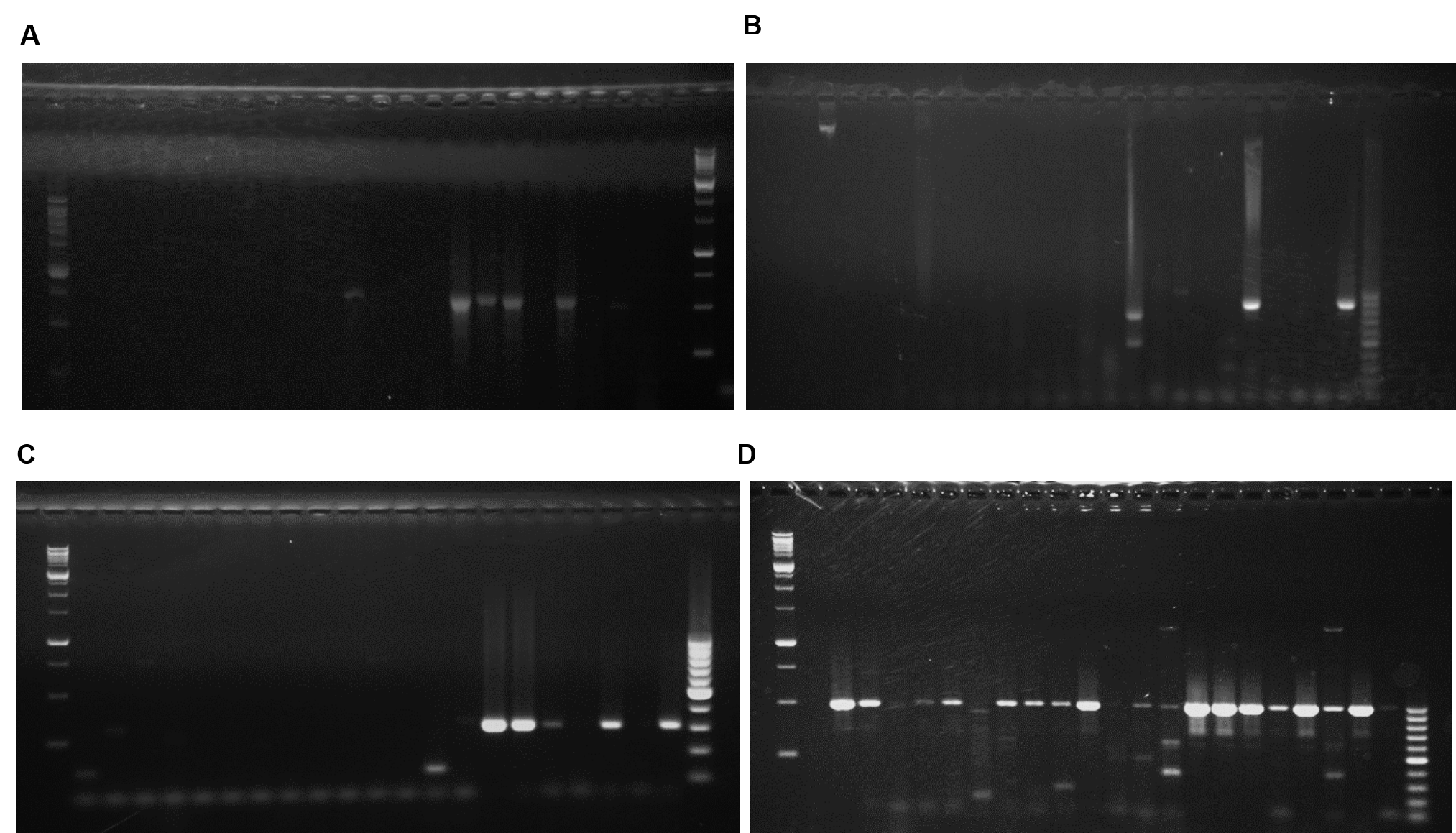

Supplement: Supplemental Information 3 [file peerj-11-15035-s003.png]
